# Short-term functional versus patient-reported outcome of the bicruciate stabilized total knee arthroplasty: prospective consecutive case series

**DOI:** 10.1186/1471-2474-15-435

**Published:** 2014-12-16

**Authors:** Matthias Christen, Emin Aghayev, Bernhard Christen

**Affiliations:** Department of Orthopaedic Surgery, Salem Spital, Schänzlistrasse 39, 3000 Bern 25, Switzerland; Institute for Evaluative Research in Medicine, University of Bern, Stauffacherstrasse 78, Bern, 3014 Switzerland

**Keywords:** Total knee arthroplasty, Knee injury and osteoarthritis outcome score, Knee society score, Journey, Bicruciate stabilized knee prosthesis

## Abstract

**Background:**

The main goals of the standard treatment for advanced symptomatic knee osteoarthritis, total knee arthroplasty (TKA), are pain reduction and restoration of knee motion.

The aim of this study was to analyse the outcome of the patient-based Knee Injury and Osteoarthritis Outcome Score (KOOS), and the surgeon-based Knee Society Score (KSS) and its Knee Score (KS) and Knee Functional Score (KFS) components after (TKA) using the Journey knee prosthesis, and to assess the correlation of these scores with range of motion (ROM).

**Methods:**

In a prospective case series study between August 1^st^ 2008 and May 31^st^ 2011, 99 patients, all operated by a single surgeon, received Journey bicruciate stabilized total knee prostheses. The female/male ratio was 53/34, the mean patient age at surgery was 68 years (range 41–83 years), and the left/right knee ratio was 55/44. The KOOS, range of motion, and KS and KFS were obtained preoperatively and at 1-year follow-up. The pre- and postoperative levels of the outcome measures were compared using the Wilcoxon signed-rank test. Correlation between ROM and patient outcomes was analysed with the Spearman coefficient.

**Results:**

All KOOS subscores improved significantly. Ninety percent of patients improved by at least the minimum clinically relevant difference of 10 points in stiffness and other symptoms, 94.5% in pain, 94.5% in activities of daily living, 84.9% in sports and recreation, and 90% in knee-related quality of life. The mean passive and active ROM improved from 122.4° (range 90-145°) and 120.4° (range 80-145°) preoperatively to 129.4° (range 90-145°) and 127.1° (range 100-145°) postoperatively. The highest correlation coefficients for ROM and KOOS were observed for the activity and pain subscores. Very low or no correlation was seen for the sport subscore.

There was a significant and clinically relevant improvement of KSS (preop/postop 112.2/174.5 points), and its KS (preop/postop 45.6/86.8 points) and KFS (preop/postop 66.6/87.8 points) components.

**Conclusions:**

The Journey bicruciate stabilized knee prosthesis showed good 1-year postoperative results in terms of both functional and patient-based outcome. However, higher knee ROM correlates only moderately with patient-based outcome, implying that functionality afforded by the Journey bicruciate TKA is not equivalent to patient satisfaction.

**Electronic supplementary material:**

The online version of this article (doi:10.1186/1471-2474-15-435) contains supplementary material, which is available to authorized users.

## Background

After the hip, the knee is the next most commonly replaced joint, with an overall incidence rate of 212 patients per 100’000 inhabitants per year in Switzerland and an even 213 patients per 100’000 inhabitants in Germany [1]. Even though patients have high expectations of pain relief and functional restoration from this treatment of end-stage knee arthritis [2–5], up to 30% of patients are not fully satisfied with their outcome [6]. Abnormal kinematics is implicated in the failure to restore the function that patients desire [6–8]. Therefore, the main goal of new prosthetic designs is not only pain relief, but also improved restoration of knee kinematics [9].

Abnormal gait and joint kinematics can interfere with recovery of function following total knee arthroplasty (TKA) [7, 8, 10]. Knee implants are characterized by various differences in design and kinematics, and the bicruciate stabilized (BCS) TKA was developed to more closely replicate normal knee kinematics and thus improve functional results compared to conventional prostheses [10–14]. Recent studies have shown that the mean range of motion (ROM) can be increased by the new TKA designs [15, 16]. BCS knees have greater active flexion compared to posterior stabilized and cruciate retaining knee arthroplasty [17]. The BCS TKA system is regarded as an optimized posterior stabilized knee prosthesis design that allows guided motion. Different in vivo fluoroscopic studies demonstrate that close to normal motion can be attained with a BCS prosthesis [9, 10, 14, 18–20].

The outcome of TKA has many dimensions that can make its assessment complex and time-consuming. Several knee scores based on patient self-assessment have been used to obtain patient perception of knee function before and after TKA [21, 22]. Clear evidence suggests that patients’ baseline levels of pain and function are highly correlated with their perception of outcome [23].

The aim of the study was to assess whether patients achieve an improved level of well-being and function after BCS TKA, and to analyse the extent to which patient-rated outcome correlates with function. We hypothesized that functional and patient-rated outcomes are well correlated.

## Methods

The study was performed in accordance with the guidelines of the local ethics committee and was approved by the local institutional review board (Salem Spital Bern). Informed consent was received from each participant.

### Study design

A prospective, consecutive case series study design was used in one orthopaedic department. During the period between August 1^st^ 2008 and May 31^st^ 2011, 113 consecutive knees in 101 patients were treated by a single surgeon [24]. Thirteen knees in the same number of patients underwent a more constrained TKA (one constrained condylar, one semi-constrained and 11 rotating hinge TKA) due to either serious medial or lateral ligament instability (>10°; n = 11), or a tibial plateau fracture (n = 2) and were excluded from the study. One further patient was excluded due to an additional supracondylar osteotomy. The Journey BCS prosthesis was implanted in 99 knees of the remaining 87 patients, who filled out questionnaires pre- and postoperatively that were analysed in the study. Twelve patients underwent two-stage, bilateral surgery, the female/male ratio was 53/34, and the mean age at surgery was 68 years (range 41–83 years). Follow-up assessment was performed 12 months after surgery.

### Measurements

Range of motion was measured preoperatively and at follow-up using a goniometer [25, 26] to obtain the maximum arcs of active and passive motion. Medial and lateral laxity were manually measured in full extension and in 30° of flexion, and were graded as stable (laxity less than 5°), lax (5 to 10°), or as very lax (>10°).

The Knee Injury and Osteoarthritis Outcome Score (KOOS) and Knee Society Score (KSS) were obtained preoperatively and one year postoperatively [24]. The KOOS is a patient-based outcome instrument (with 100 as the best possible and 0 as the worst possible outcome) that evaluates short- and long-term symptoms and function in persons with knee injury and osteoarthritis. It has been used for follow-up of orthopaedic interventions such as anterior cruciate ligament reconstruction, meniscectomy, and total knee arthroplasty [21]. The questionnaire has five separately scored subscales: stiffness and other symptoms, pain, activities of daily living, function in sport and recreation, and knee-related quality of life. To ensure validity of the KOOS, the Western Ontario and McMaster Universities Arthritis Index (WOMAC) was included in the KOOS and it is possible to extract WOMAC scores from the KOOS [21, 27]. The translated and validated German version of the KOOS instrument was used in the study [28].

The KSS, which is one of the most frequently used measures in knee orthopaedics, is subdivided into two scores, the Knee Score (KS) and the Knee Functional Score (KFS) [22]. The KS rates pain, stability, and range of motion, allotting a maximum 100 points to a well aligned knee with no pain, 125° of flexion, and negligible antero-posterior and medio-lateral instability. The KFS rates the patient’s ability to walk and climb stairs, also with a maximum score of 100 points attained by walking an unlimited distance without aids and climbing stairs without the use of a handrail [22].

ROM was correlated with the patient-reported factors: stiffness and other symptoms, pain, activities of daily living, sports and recreational activities, and quality of life [24].

### Surgical technique

All knees were operated with the tibia-first osteotomy technique. The first 14 TKA (14.1%) were performed using standard instrumentation with extramedullary tibial osteotomy and intramedullary distal femoral osteotomy alignment. For the next 85 TKA (85.9%), computed tomography navigation (PI Galileo, Smith & Nephew, Aarau, Switzerland) was used to define the tibial and distal femoral osteotomies. The main femoral osteotomies in all TKA were performed with the balanced-gap technique in full extension and 90° of flexion. The knees were balanced in extension by an in-tube release on the concave side in case of fixed deformity before the distal femoral osteotomy. The osteotomy was performed as soon as an equal medial and lateral force applied by a tensioner (Smith & Nephew, Memphis, USA) led to a neutral mechanical axis. This was clinically controlled according to preoperative planning in the first group and by the navigation system in the second. The femoral rotation was defined using the tensioner, again in 90° of flexion, applying an equal force on the medial and lateral side. Then the femur was cut anteriorly and posteriorly parallel to the tibial osteotomy, which is called the balanced gap technique. To minimize errors, the classical bony landmarks (trans-epicondylar and posterior condylar lines) were included as a safety check for the ligament balancing. The patella was mostly resurfaced with an inlay technique using reamers of appropriate sizes. All components were cemented in one step starting with the tibial component first, then the femoral one, and ending with the patella if resurfaced. With the prosthesis in place, the optimal thickness of the polyethylene insert was determined using different trials. The system allows for 1 mm increments for insert thicknesses between 9 and 11 mm, and 2 mm increments in thicker inserts. An insert thickness of 9 mm was used in 45.5% of the 99 knees, 10 mm in 27.3%, 11 mm in 24.2%, and a 13 mm in 3.0%.

The main focus in all knees was to reach a perfectly stable knee in extension, and in 30° and 90° of flexion on the medial side to guarantee optimal medial pivoting.

### Statistical methods

The pre- and postoperative scores and ROM were compared using the Wilcoxon signed-rank test. Correlation of KOOS and ROM was analysed using the Spearman rank-order correlation coefficient. The minimum clinically relevant difference (MCRD) for each KOOS subscore was set at 10 points as suggested by Roos et al. [21]. The proportion of patients who achieved the MCRD for each of the subscores was calculated.

All statistical analyses were conducted using SAS 9.4 (SAS Institute Inc., Cary, NC) with α = .05.

## Results

Mean preoperative KOOS subscores for stiffness and other symptoms, pain, activities of daily living, sport and recreation, and knee-related quality of life were 44.4, 38.0, 42.8, 24.9 and 21.7, respectively. At 1-year the respective follow-up scores increased to 80.0, 84.2, 85.6, 73.2, and 74.4. All the differences were significant (p < .001 for all subscores).

Ninety percent of patients reached or exceeded the MCRD for stiffness and other symptoms, 94.5% for pain, 94.5% for activities of daily living, 84.9% for sport and recreation, and 90.0% for knee-related quality of life.

There was a significant and clinically relevant improvement of KSS from 112.2 points (range 47–164) preoperatively to 174.5 points (range 85–200) postoperatively. The KS and KFS components improved from 45.6 points (range 25–84) and 66.6 points (range 10–100) to 86.8 points (range 45–100) and 87.8 points (range 35–100), respectively.

The mean preoperative passive and active ROM were 122.4° (range 90-145°) and 120.4° (range 80-145°). They both improved significantly to 129.4° (range 90-145°) and 127.1° (range 100-145°), respectively (p < .001).

Low to moderate correlation was observed between the KOOS subscores and passive and active ROM (range 0.16 - 0.45) (Table 1). The highest of the correlation coefficients observed for the KOOS subscores were for passive ROM and stiffness and other symptoms (r = 0.36) and pain (r = 0.35). Relatively higher, moderate correlation was seen between the KS score and passive (r = 0.45) and active (r = 0.37) ROM.

**Table 1:** **Correlation between ROM and KOOS and KSS scores**

Scores	Spearman correlation coefficient	
	Passive ROM	Active ROM
KOOS subscore: Stiffness and symptoms	0.36*	0.25*
KOOS subscore: Pain	0.35*	0.28*
KOOS subscore: Activity	0.31*	0.21*
KOOS subscore: Sport	0.27*	0.18
KOOS subscore: Quality of life	0.24*	0.16
KOOS complete score	0.33*	0.23*
KSS subscore: KS	0.45*	0.37*
KSS subscore: KFS	0.28*	0.23*
KSS complete score	0.40*	0.33*

Note: * - p < .05.

Regarding medial and lateral knee stability, 97.8% of patients had both preoperative and postoperative laxity below 5°, and the remaining 2.2% patients between 5 and 10°.

## Discussion

Our study shows good to excellent results in all five KOOS subscores, which improved on follow-up by at least 36 to as many as 53 points. Based on the ROM measurements, the postoperative function of patients with the Journey BCS prosthesis is close to the physiological knee. A review by Chiu et al. [29] reported a postoperative ROM of 95°-115°. In a comparison of two guided motion knee systems, Mugnai et al. reported a mean ROM of 115° [30]. Our study found mean postoperative active and passive ROM of 127° and 129°.

The Knee Society scores were similarly improved. Good to excellent postoperative KS and KFS results also have been reported in other TKA studies. Brander et al. reported mean preoperative KSS, KS, and KFS values of 90.6, 45.8, and 45.0 points, and respective postoperative values 161.9, 87.0 and 76.0 at 5-year follow-up [31]. The pre- to postoperative change of KS in the Brander et al. study, 41.2, is exactly the same as that in our study. However, the pre- to postoperative change in KFS was higher in the Brander study than in ours (31.0 vs. 21.2). This is clearly due to the fact that patients in our study had a higher mean preoperative KFS than did those in the Brander et al. study (66.6 vs. 45.0). A similar pre- to postoperative KS change of 34.0 and a KFS change between those observed in our study and that of Brander et al., 27.0, was reported by Becker et al. in a study with follow-up at eight months [32].

In a 2011 literature review, Bonnin et al. reported a mean postoperative pain score of 86.0 points, measured using the WOMAC questionnaire, as acceptable residual pain after a well-performed and uncomplicated TKA [33]. Argenson et al. reported mean KOOS pain score results of 82.0 points 2–4 years after TKA using high flexion mobile bearing posterior-stabilized knee implants [34]. We observed similar postoperative WOMAC pain score results of 84.2 points in our study.

Despite these convincing results regarding functional and patient-based outcomes, higher complication rates were recently reported for Journey BCS knees compared to other, established total knee systems. Most of the observed complications can be related to the prosthesis design [35].

Only low to moderate correlation was observed in our study between the KOOS subscores and passive and active ROM. The highest subscore correlation coefficients were observed for stiffness and other symptoms, and pain, which are the first indicators of the musculoskeletal disorder that is knee arthritis. The TKA related limitations in patients’ general activity or sport clearly play a secondary role, and overall quality of life may not be as affected as the joint- and disease specific indicators. Among the factors involved in patient satisfaction, perception of a certain ROM as a satisfying outcome may potentially be influenced by other factors such as the ROM of the contralateral knee or the function of other lower extremity joints; neither of the knee scores we employed takes into account the status of the contralateral knee. A high ROM of the treated knee also may not automatically mean low disability; a ROM over 120° increases the risk of knee instability and does not benefit the patient. Other factors as well, including comorbidities and overall satisfaction with the treatment, may influence patient perception of disability.

Although both the KSS score and passive and active range of motion represent knee function, their correlation was moderate. As anticipated, the KS subscore incorporating pain, stability perception, and range of motion had a higher correlation with physician-assessed range of motion than did the ability to walk and climb stairs. In all instances, correlation coefficients of KOOS and KSS scores with passive range of motion were higher than those with active range of motion. This could suggest greater accuracy in the assessment of the passive ROM than active ROM, which may more often be affected by various musculoskeletal or other limitations.

### Limitations

This study relies upon a prospective case series, but it benefits from having had a single surgeon who was experienced in the implantation of the Journey BCS prosthesis. However, its consistency is somewhat diminished by the fact that the first 14 TKA (14.1%) were performed using standard instrumentation for the extramedullary tibial and intramedullary femoral osteotomies, while the remaining 85 TKA (85.9%) tibial and femoral osteotomies were performed under computer tomography navigation. It should be borne in mind as well that this is a short-term study, and longer-term studies have reported continuing decline in KOOS subscores through five years postoperatively [31, 36].

## Conclusions

The Journey bicruciate stabilized knee showed good to excellent postoperative range of motion and knee joint stability, and good to excellent outcome when assessed by both physician- and patient-based instruments. While these clinical results are comparable to those reported in other studies of this prosthesis, functional range of motion and patient-rated KOOS subscores are not, as we hypothesized, well correlated; the correlation is low to moderate. In other words, functional outcome is not a proxy for patient satisfaction.

## Authors’ original submitted files for images

Below are the links to the authors’ original submitted files for images. Authors’ original file for figure 1

## Abbreviations

BCS: Bicruciate stabilized
KFS: Knee functional score
KOOS: Knee injury and osteoarthritis outcome score
KS: Knee score
KSS: Knee society score
MCRD: Minimum clinically relevant difference
ROM: Range of motion
TKA: Total knee arthroplasty
WOMAC: Western Ontario and Mcmaster Universities Arthritis Index.
