# Supplementary figures and images for: Short-term functional versus patient-reported outcome of the bicruciate stabilized total knee arthroplasty: prospective consecutive case series
Source: BMC Musculoskelet Disord. 2014 Dec 16;15:435. doi: 10.1186/1471-2474-15-435 (PMC4300849; doi:10.1186/1471-2474-15-435)

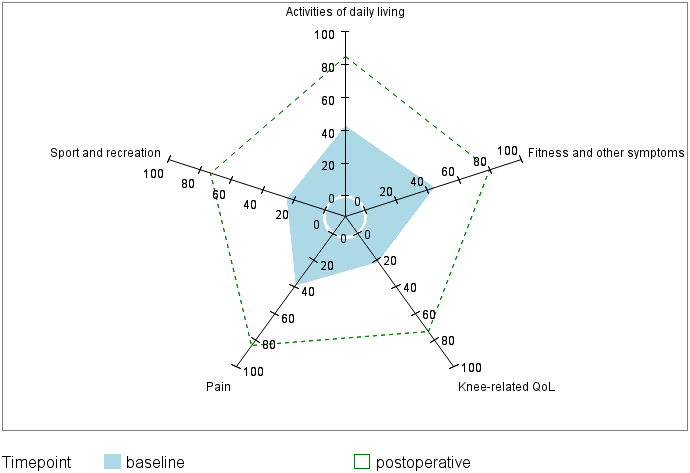

Supplement: Supplementary file 1 — Authors’ original file for figure 1 [file 12891_2014_2366_MOESM1_ESM.tif]
